# “I Got My Trophy”: The Story of Implementing a Neuro-Oncology Exercise Program from the Patient and Caregiver Lens—A Qualitative Study †

**DOI:** 10.3390/curroncol32020111

**Published:** 2025-02-16

**Authors:** Julia T. Daun, Mannat Bansal, Randall L. Iversen, Meghan H. McDonough, Gloria Roldan Urgoiti, Tana Dhruva, Emma McLaughlin, Lauren C. Capozzi, Jacob C. Easaw, Margaret L. McNeely, George J. Francis, S. Nicole Culos-Reed

**Affiliations:** 1 Faculty of Kinesiology, University of Calgary, Calgary, AB T2N 1N4, Canada; mannat.bansal@ucalgary.ca (M.B.); meghan.mcdonough@ucalgary.ca (M.H.M.); tana.dhruva@ucalgary.ca (T.D.); emma.mclaughlin1@ucalgary.ca (E.M.); nculosre@ucalgary.ca (S.N.C.-R.); 2 Patient Partner, Calgary, AB T2N 1N4, Canada; randy.iversen@shaw.ca; 3 Department of Medical Oncology, Arthur J.E. Child Comprehensive Cancer Centre, Alberta Health Services, Calgary, AB T2N 4N1, Canada; gloria.roldanurgoiti@albertahealthservices.ca; 4 Department of Clinical Neurosciences, Cumming School of Medicine, University of Calgary, Calgary, AB T2N 4N1, Canada; lcapozzi@ucalgary.ca (L.C.C.); george.francis@ucalgary.ca (G.J.F.); 5 Department of Medical Oncology, Cross Cancer Institute, Edmonton, AB T6G 1Z2, Canada; jay.easaw@albertahealthservices.ca; 6 Department of Physical Therapy, University of Alberta, Edmonton, AB T6G 2G4, Canada; mmcneely@ualberta.ca; 7 Supportive Care and Patient Experience, Cancer Care Alberta, Edmonton, AB T5J 3E4, Canada; 8 Department of Oncology, Cumming School of Medicine, University of Calgary, Calgary, AB T2N 4N1, Canada; 9 Department of Psychosocial Resources, Arthur J.E. Child Comprehensive Cancer Centre, Alberta Health Services, Calgary, AB T2N 4N1, Canada

**Keywords:** neuro-oncology, exercise oncology, implementation science, qualitative research, patient-oriented research, patient-centred care, health behaviour change, tailored interventions

## Abstract

The purpose of this study was to gather patient and caregiver perspectives of adult neuro-oncology patients participating in a 12-week exercise program (i.e., the Alberta Cancer Exercise-Neuro-Oncology; ACE-Neuro study). Patients and their caregivers were invited to participate in semi-structured interviews across study delivery. A qualitative photo elicitation methodology within a patient-oriented research approach was used. Interpretive description and a constructivist philosophy guided the investigation, analysis, and dissemination of findings. A patient partner was included as a member of the research team. *N* = 51 patients completed the ACE-Neuro study, of which 28 patients and nine caregivers participated in interviews (*n* = 37). Working with the patient partner, five themes were created and are presented as a story of neuro-oncology patients on their journey to accessing and participating in ACE-Neuro: (1) The Exposition: I Have Cancer…Now What?; (2) The Rising Action: Trials and Triumphs of Participation; (3) The Pivotal Moment: It’s More Than Exercise; (4) The Resolution: Tailored Not Templated…The Ideal Program for Me; and (5) The Epilogue: Key Factors for Sustained Delivery. The findings from this work address the lack of qualitative exploration for understanding the neuro-oncology exercise experience and will inform the sustainable implementation of programming to meet patients’ needs.

## 1. Introduction

Implementing patient-centred and tailored exercise programming as part of neuro-oncology care is a growing need. Though evidence supports the safety, feasibility, and benefits of exercise for this population [1], neuro-oncology patients continue to face barriers accessing and engaging in exercise programming [2]. These barriers exist in part due to the challenges neuro-oncology patients experience, including difficult and intensive treatment timelines and high symptom burden (e.g., seizures, hemiparesis, cognitive decline, and aphasia). These factors often impact an individual’s health-related quality of life and ability to adhere to a fixed-schedule exercise program [3,4,5,6]. In addition, the lack of a systematic and streamlined pathway for referral to community-based exercise programming has contributed to these barriers and thus the low representation of neuro-oncology patients in exercise oncology programs [7]. In Alberta, a transdisciplinary approach (i.e., collaboration across multiple healthcare disciplines, including oncology, rehabilitation medicine, and exercise physiology) has advanced the standard of neuro-oncology care to include exercise across the treatment timeline (i.e., pre-treatment, during, and post-treatment) [8]. The Alberta Cancer Exercise-Neuro-Oncology (i.e., ACE-Neuro) study has built a referral pathway via the electronic medical record [9] to tailored, flexible, and multimodal exercise programming that is supported by health coaching [8]. To support the sustainability of ACE-Neuro and ensure that it meets the unique needs of the neuro-oncology population, gathering patient and caregiver feedback is critical [10].

Specifically, obtaining feedback from these key end-user groups is essential for assessing the implementation logistics (i.e., feasibility, delivery, training of interventionists) and sustainability (i.e., continued delivery over time) [11] of exercise interventions from the patient’s perspective. Across other tumour groups, such as breast, prostate, colorectal, gynecologic, lung, blood, and head and neck cancers, patients and caregivers have been engaged in the research process [12,13,14,15,16], leading to the development and delivery of effective and patient-centred exercise oncology programs. Key insights from this work may be relevant to neuro-oncology. For example, strong evidence exists for understanding the role of exercise for improving quality of life and overall physical and psychosocial well-being, attitudes towards exercise, barriers and facilitators to exercise, preferences for timing and format of exercise across treatment stages, support needs, and considerations for sustainable delivery of programming [12,13,14,15,16].

Nevertheless, while the evidence base for patient-informed exercise oncology programming is well-established in these populations, gaps remain for applying these findings to neuro-oncology, given the distinct and complex experiences of individuals living with and beyond brain cancer. Further, little work has been done to develop exercise programming with active involvement of neuro-oncology patients and caregivers, making it difficult to understand the neuro-oncology experience within the context of exercise and implementing interventions that are truly tailored to patient needs. The literature to date has mainly focused on the experience of living with a brain tumour and supportive care needs [17,18,19,20]. Current gaps in knowledge include understanding neuro-oncology patients’ perceptions of exercise across the care continuum, motivations and challenges to participation in exercise, and needs to support adherence. Direct input from patients and caregivers can thus answer these questions and inform the design of a program that is flexible and adaptable to individual patient needs. Without this feedback, there is a risk of developing interventions that are inaccessible and ineffective for improving patient outcomes specific to the neuro-oncology care continuum [21]. Given that prior work has overlooked this important research area, the novelty of the present study is grounded in patient-centred insights to inform tailored neuro-oncology exercise interventions.

The purpose of this study was to understand the experiences of patients and their caregivers within the ACE-Neuro study. Specifically, the goal was to gain insights into how the 12-week exercise program was implemented, including the referral process, exercise intervention, and completion of measures, as well as needs for continued delivery.

## 2. Materials and Methods

### 2.1. Participants and Setting

This study was approved by the University of Calgary Health Research Ethics Board of Alberta—Cancer Committee (HREBA.CC-20-0322). As part of the ACE-Neuro study [8], eligible participants included adults ≥18 years who were diagnosed with a primary brain tumour, treated at one of two tertiary cancer centres in Alberta, and able to consent in English. Patients were referred to this study either via (1) the electronic medical record (Calgary site) or (2) self-referral (e.g., brochure, word of mouth; both sites). Participants were in a 12-week tailored exercise intervention that included one-on-one and group-based exercise sessions and weekly health coaching sessions to support behaviour change, delivered either in-person, online, or both (i.e., hybrid). Participants completed baseline and 12-week measures of patient-reported outcomes (online) and functional fitness (online or in-person), as well as measures of objective physical activity across the full 12 weeks via a Garmin activity tracker [22]. All protocol details have been previously published [8].

### 2.2. Qualitative Methodology and Patient Engagement

Within this study, a qualitative photo elicitation methodology was used. While exercise oncology research has used varied qualitative approaches to gather participant perspectives, interviews are often used to provide depth in the feedback that captures the human emotion and experiences beyond typical self-reported outcome measures [23]. Photo elicitation includes presenting photographs to participants during an interview to evoke information, emotions, and memories related to participation [24]. By prompting reflections through visual cues, photo elicitation may help to reinforce the role of movement across the neuro-oncology care continuum, especially for patients who face challenges with memory, recall, and word finding. Further, engaging patients as partners beyond the interviews, including during data analysis and dissemination, can further enrich findings by ensuring the research process remains grounded in and relevant to the lived experiences of patients [25]. Such a collaborative approach is valuable for building trust and creating shared visions of supportive cancer care among patients and caregivers, researchers, and clinical teams, ultimately leading to more effective and patient-centred care [25]. A patient partner (third author—R.L.I.) was engaged throughout the study process. Beyond participating in the exercise intervention, the patient partner was invited to analyze and disseminate the research findings.

From study conceptualization, through investigation, and to analysis and dissemination of findings, an interpretive description methodology [26] and constructivist philosophy [27] were used, both of which are relevant for applied health research questions. The theoretical scaffolding of interpretive description within the present study included the consideration of the wider field of exercise oncology, the unique needs for working with individuals living with and beyond brain cancer, theories of behaviour change for supporting adherence to exercise, and implementation frameworks for understanding implementation needs in applied research settings.

### 2.3. Study Procedures

If participants opted to partake in photo elicitation, photographs were taken either pre-, during, or post-exercise sessions (1) by the lead author, (2) the exercise professional working with the participant, or (3) the participant or caregiver. Supplementary File S1 presents the photo elicitation protocol. Photos could include the one-on-one or group-based exercise sessions, exercise equipment, a participant’s at-home exercise set-up, or any person, place, or item that reminded them of ACE-Neuro (e.g., their caregiver, documents such as the ACE-Neuro welcome package). During the interviews, the participants who engaged in photo elicitation were presented their photographs alongside additional photo elicitation-specific questions to elicit their memories and feelings of ACE-Neuro.

Semi-structured interviews were conducted by the lead author (J.T.D.) with participants, and when relevant their caregivers via purposive sampling of the larger trial. Sampling was based on the study team’s review of participants’ tumour type, age, and self-identified gender to ensure varied perspectives were gathered. Cognizant of participant burden, this study was initially designed to include only one interview post-intervention. Partway through this study, it was clear that some participants might experience more challenges with the duration of rather than the number of interviews. Thus, the research team decided to start offering participants the option to complete either (1) one longer interview post-intervention or (2) two shorter interviews pre- (i.e., at the time of the baseline assessment) and post-intervention. Interviews were conducted either in-person or remotely via ZOOM^TM^ and were scheduled for 15–60 min. All interviews were recorded via ZOOM^TM^. The COM-B behaviour change framework, which identifies that examining participant capabilities, opportunities, motivations, and behaviours are all important topics of inquiry for understanding health behaviour, was used to inform the development of the interview guide, which is presented in Supplementary File S2. One open-ended question from a post-intervention satisfaction survey was included in the qualitative analysis. Quantitative survey responses are reported separately [28].

It was important to consider the needs of neuro-oncology patients (e.g., emotional distress, fatigue, cognitive overload) when designing this study so as to not overburden participants. Therefore, the research team (1) received feedback on interview guides from clinicians, researchers, and qualitative experts adept in the field of neuro- and exercise-oncology, (2) practised interview delivery with colleagues for feedback on interviews style and active and empathetic listening skills, and (3) made a plan to refer participants to psychosocial services in the event that they experienced emotional distress.

### 2.4. Analysis

Interviews were transcribed verbatim using ExpressScribe Version 13 transcription software by research assistants and checked against the interview recordings by the lead author (J.T.D.) [29]. The lead author then familiarized herself with one transcript and associated interview notes at a time prior to coding. The coding process involved re-reading each transcript, identifying and labelling segments of text that expressed meaning related to the research question, and coding text with similar meaning across transcripts. The development of codes was supported by deep reflection, stepping back from the data, and critical discussion with the second (M.B.) and fourth (M.H.M.) authors [30]. Following coding, the lead (J.T.D.), second (M.B.), and third (R.L.I.; patient partner) authors created representative themes. This iterative process included reviewing and mapping codes into groups and a collaborative conversation during a six-hour in-person analysis day (Supplementary File S3). To enhance study rigour, the four principles distinct to interpretive description were followed during the study [26]. Specifically, the lead author (J.T.D.) referred to the principles of interpretive description throughout this study, had prolonged engagement with the study and its participants, maintained an audit trail of all study decisions and changes, and kept a reflexivity journal to log personal perspectives, reflections, and biases throughout the study.

### 2.5. Researcher Positionality

Detailing of researcher positionality focuses primarily on the first three authors most involved in conducting the analysis. The lead author is a PhD candidate coordinating the ACE-Neuro study. She identifies as a 31-year-old, cisgender female, and second-generation Polish Canadian. She is younger than most of the sample and is not living with and beyond cancer. She has worked in exercise oncology for over eleven years as a Clinical Exercise Physiologist, graduate student trainee, and health coach. She has lost many friends and family to cancer, including brain cancer, and is also a cancer caregiver. She thus has a comprehensive understanding of exercise oncology and the cancer experience from a caregiver lens and has a deep-rooted passion to make wellness a part of supportive cancer care.

The second author (M.B.) is a 26-year-old, cisgender female, second-generation Canadian born to Canadian immigrants of Indian origin. She has seen multiple family members diagnosed with various cancers, including glioblastoma. She watched their journeys through the Canadian healthcare system and was witness to their lack of access to supportive care services, including exercise oncology resources. She is a PhD candidate and has been an exercise oncology instructor for the past four years. These experiences were foundational in her passion for making exercise oncology resources accessible for all.

The third (R.L.I.) author is an 84-year-old retired English teacher living with brain cancer. He participated in the ACE-Neuro study and thus has first-hand experience with the neuro-oncology diagnosis, treatment continuum, and intervention itself. He has right-sided paralysis and cognitive challenges, both of which impact his day-to-day function. He values the role of exercise in managing his recovery from surgery and radiation and thus has a vested interest in engaging in the current work.

The remainder of the authors involved in this work comprise a transdisciplinary team of researchers and clinicians with professional expertise in the areas of qualitative research, exercise oncology, exercise psychology, and neuro-oncology care.

## 3. Results

### 3.1. Interview Results

From April 2022 to December 2023, *n* = 51 neuro-oncology patients completed the 12-week ACE-Neuro intervention with an adherence rate of 89.7% [28]. A total of 37 participants completed interviews, including 28 patients and nine caregivers. Eight of the caregivers participated in a dyadic interview with the patient, and in one case, just the caregiver was present due to their spouse’s (i.e., the patient’s) death. The patient passed away during the study, and the caregiver was able to speak about their knowledge of the patient’s experience with the exercise program. Twenty-nine patients and caregivers were from Calgary (main study site), and *n* = 8 were from Edmonton. One patient and two patient-caregiver dyads chose to partake in the two interview timepoints (i.e., the first one at the time of the baseline fitness assessment and the second one post-intervention) and the remainder (i.e., n = 19 patients alone, *n* = 6 dyads, *n* = 1 caregiver alone) chose the one post-intervention interview. Of the 32 total interviews, *n* = 6 took place in person, and *n* = 26 were via ZOOM^TM^. Average interview length was 44:08 ± 19:21 min (range: 7:39–98:40 min). *N* = 25 participated in photo elicitation. The three who did not either preferred not to take or have photos taken or forgot to. For the caregiver-only interview, photo elicitation was completed with the patient during the program, but given the patient had recently passed away, it was decided by the research team to not share the photos during the interview.

### 3.2. Demographic and Clinical Characteristics of the Sample

Table 1 shows the complete clinical and demographic profile of patients. Sixteen patients were female (57.1%), and twelve were male. Patients were mainly middle-aged (51.4 ± 11.7 years, range 32–81) and averaged 58.5 ± 84.1 months since diagnosis (1–290-month range). Glioblastoma was the most common diagnosis (*n* = 9). Over half of the sample (19/28, 67.9%) had undergone a combination of surgery, chemotherapy, and radiation treatments. Patients were of high socioeconomic background, with many completing higher education at the bachelor’s (*n* = 13) and master’s or doctorate levels (*n* = 8). Of the *n* = 19 reporting income, nine reported earnings exceeding >CAD 100,000, surpassing the provincial median [31], while the remaining ten patients reported incomes ranging from CAD 20,000 to CAD 99,000. Most were married (*n* = 22) and either on long-term disability (*n* = 12), short-term disability (*n* = 2), or were temporarily unemployed (*n* = 2). Only six patients reported working part-time (*n* = 1), full-time (*n* = 3), or as a homemaker (*n* = 2); and *n* = 6 were retired. Patients self-identified as White (*n* = 22), Indigenous (*n* = 2), South Asian (*n* = 2), East Asian (*n* = 1), and Southeast Asian (*n* = 1). Caregivers included spouses (*n* = 7), parents (*n* = 1), and children (*n* = 1) and were primarily female (*n* = 6, 66.7%) (Table 2).

### 3.3. The Story of Implementing ACE-Neuro—Resultant Themes

**Figure 1:**
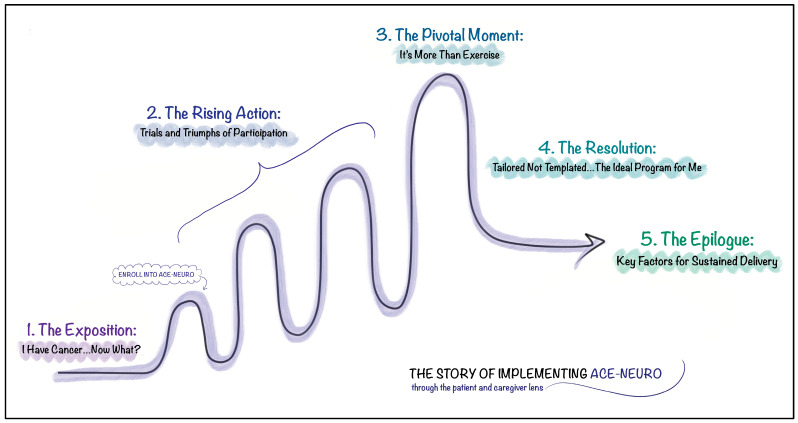
The story of implementing ACE-Neuro.

Working with the patient partner, a retired English teacher, the implementation of ACE-Neuro was discussed by him as a story in five acts. Thus, the results are presented using this analogy. Analysis led to the development of five themes that described the story of individuals living with and beyond brain cancer (i.e., the protagonist) on their journey to wellness (i.e., via ACE-Neuro). These themes were then organized into a story plot diagram to convey the protagonist’s journey across the five acts (Figure 1): (1) The Exposition: I Have Cancer…Now What?; (2) The Rising Action: Trials and Triumphs of Participation; (3) The Pivotal Moment: It’s More Than Exercise; (4) The Resolution: Tailored Not Templated…The Ideal Program for Me; and (5) The Epilogue: Key Factors for Sustained Delivery (Figure 1). Each theme is described and illustrated using example quotations. Quotations are labelled with the participant’s role (i.e., patient or caregiver); identifying number of the caregiver-patient dyad; self-identified sex; and for patients, their age and type of cancer and, for caregivers, their relationship to the patient. Additional exemplary quotes are presented in Supplementary File S4.

**Theme 1—The Exposition: I Have Cancer…Now What?** 

The ACE-Neuro story begins with the cornerstone moment when patients were diagnosed with brain cancer and their world as they knew it changed. For many, their diagnosis became their worst nightmare.

*That’s the horror diagnosis in every movie, right. You’ve got a brain tumor […] every single movie, you know, that’s what they use…that’s the scariest thing can happen to someone.* 

(Caregiver no. 40, female, spouse).

Patients spoke to their experiences navigating a life-altering diagnosis and what it is like living with a brain tumour. Patients experienced feelings of fear, uncertainty, loss of hope, loss of independence, and loss of control. Patients’ loss of independence and control were especially relevant to their physical activity levels as their diagnoses, treatments, and side effects impacted their abilities to perform activities of daily living (e.g., dressing, eating, ambulating, toileting), instrumental activities of daily living (e.g., housework, transportation, managing finances), engage in the exercises they previously enjoyed, and work in their respective professions. Though all patients felt the impact of their disease, there was variation in how the “before and after” of a brain tumour diagnosis was experienced. Those who were retired or in a later stage of life experienced a sense of resignation or focus on managing symptoms in a way that allowed them to maintain some semblance of routine. In contrast, younger patients faced challenges more relevant to maintaining their careers, engaging in social activities and self-exploration, and raising their families or planning to start them. Further, some participants had a more pragmatic approach to dealing with their new diagnosis, focusing on practical adaptations to their routines and physical limitations (e.g., installing railings in their home for balance support), while others experienced a more emotional response, grappling with feelings of loss (e.g., struggling to accept their inability to move their bodies like they once could).

*I’ve always been active my whole life. I’ve ran 2 marathons, 12 half marathons, involved in sports my whole life […] So, to go from that to not being able to walk up the stairs, it’s… very discouraging, right? My husband has to walk behind me when I walk up the stairs because I don’t know if I’m gonna make it up the stairs […] It’s frustrating, not being able to move my body in the ways that I want to. That’s been a big part of the problem. Being so tired that you can’t move. Right, your body in the way that you want to or frustrated in things […] I can’t go anywhere, I can’t do anything.* 

(Patient no. 40, female, 52, presumed glioma).

In grieving their old lives and navigating their new normal, many patients and caregivers spoke to the role caregivers undertake by carrying the burden of care for many activities like driving, house chores, and simply ensuring the day-to-day safety of patients. Caregivers became inseparable from their loved ones with cancer—almost like a lifeline of constant support, intricately involved across all phases of the cancer journey. For those without a caregiver, the challenges of their diagnosis were even more pronounced, as patients faced a stressful time in their lives without additional support, increased isolation (especially during the COVID-19 pandemic), and struggled to manage daily tasks and medical needs on their own, which further exacerbated feelings of vulnerability.

*Well, for one thing, I do all the driving. He’s never given his license up… [but] I don’t even let him drive […] I always have to be with him […] I’m always concerned about his balance […] I’m always concerned about, you know, look there’s a curb there, you know those sorts of things. Yeah, I’d say you know, I had to be a Siamese twin, really. Attached, you know.* 

(Caregiver no. 40, female, spouse).

In terms of exercise history, some patients had never engaged in formal exercise, while others had ample experience going to the gym or participating in structured activities. Regardless of movement background, many were compelled by their loss of function and/or need for intensive support from their caregivers to join ACE-Neuro and improve their physical and psychosocial well-being. For many, their caregivers and healthcare providers were also a source of motivation for enrolling into the program. Healthcare providers were seen as a key motivator for participation—as trusted experts whose endorsement in the program felt credible and essential for supporting management of disease-related symptoms and improving quality of life. In facing a challenging diagnosis, many patients embraced exercise as a stepping stone towards beginning their journey to wellness.

**Theme 2—The Rising Action: Trials and Triumphs of Participation** 

Upon enrolling into ACE-Neuro, patients and caregivers embarked on a journey of trials (i.e., challenges) and triumphs (i.e., facilitators) to engaging in and adhering to the 12-week exercise program and its assessments. Unsurprisingly, this path was distinct for each participant, with varying experiences with recruitment, participation, and completing assessments. Recruitment to the study was mainly described as a seamless process, with participants appreciating the organization and timing of referral and feeling like the ACE-Neuro staff worked cohesively as a team. On the other hand, a few participants had challenges receiving information about this study. These participants heard about the study on their own (e.g., word of mouth, study brochure, own research) instead of from their healthcare providers. This lack of communication within the clinical setting was described as a disappointing obstacle for accessing wellness resources.

*I actually looked for the study on my own […] in hindsight, now what kind of disappoints me, is that in my follow up clinic, folks never mentioned the study to me […] Now that I know that that’s often the way that patients do get to you….It’s just I’m not sure what happened to me, why I got left out.* 

(Patient no. 65, female, 40, astrocytoma).

Once recruited, individuals were tasked with completing assessments (i.e., patient-reported outcomes, functional fitness, and tracking objective physical activity), which was viewed as manageable for a majority of participants, with both the types of measures and timing of them (i.e., pre- and post-intervention) perceived as appropriate. Some participants, especially those further past their initial diagnosis or in a “maintenance” phase of exercise behaviour change [33], valued the assessments as a means to reflect on their current health status and track their progress. For others who were undergoing active treatment, had more significant disease-related limitations (e.g., vision loss), or were just starting to build or rebuild their exercise habits, completing certain assessments was challenging.

*The fitness tests are important for us to know if we are improving or, you know, if there are any challenges that we are undergoing right now, and doing those assessments is also kind of like monitoring […] what’s the progress of…what the program is giving us or like the effect on us.* 

(Patient no. 4, female, 53, meningioma).

Some participants also found the questionnaires repetitive, long, and that they did not always capture the true experience of their limitations. For example, for those who were retired or on short term disability, answering questions related to their abilities to work were seen as irrelevant, yet patients did not have an option to provide a more nuanced response that reflected their unique circumstances. The Garmin activity tracker worn during the study imposed a significant burden for some participants as they did not feel they had capacity for dealing with it. Some participants felt that the watch was hard to see (i.e., the screen with daily steps was too small), hard to remember to charge, or would not accurately track their daily movement. Others really enjoyed the immediate feedback and accountability provided by the tracker and went on to purchase their own post-study completion.

*Oh, I hated it [the Garmin]. I’d wear it all day as I was told to, and I’d walk out of working out…and it would say ‘get up and move’ and it’s like…seriously? I just had an hour and a half working out and now it’s telling me to work out. So, the prompt… kept sending me those messages like ‘you need to sleep, you need to exercise, you need to go for a walk,’ but it didn’t seem to have any correspondence to what I was actually doing.* 

(Patient no. 6, female, 42, glioblastoma).

With regard to program delivery, having a supervised structure led by trained exercise professionals provided accountability and support. The approach to delivery that included scheduling based on preferred time of day (e.g., when participants had the most energy) as well as rescheduling of exercise sessions was also appreciated. Many patients experienced last-minute changes to their treatment schedules and symptoms that changed daily and varied in severity and thus valued the ability to have autonomy over their exercise schedule and ultimately felt supported to adhere to programming. The role of caregivers was also important for participants, as many could not participate without support from their loved ones.

*I could look at my week and plan the two days no problem but that morning, sometimes life changes and so to be able to pick up the phone and be like ‘oh sorry I can’t be there at noon’ um was really important and then we would find another day in that week to reschedule […] it was really great to have that flexibility to rearrange if I couldn’t make it to the class right then I could find a spot for a one-on-one session.* 

(Patient no. 7, female, 53, glioblastoma).

Participants with greater symptom burden or who were earlier in their exercise behaviour change journey faced more challenges when engaging in study components. These challenges were not merely seen as obstacles to overcome but rather part of an ongoing process for understanding individual capabilities and opportunities in relation to exercise across the neuro-oncology care continuum. The flexible and supportive approach to study delivery proved to be key facilitators in navigating the trials and triumphs to adhering to ACE-Neuro.

**Theme 3—The Pivotal Moment: It’s More Than Exercise** 

With participation, patients and caregivers came to see that the program offered more than “just an exercise prescription.” First, patients and caregivers felt that the program helped them recognize exercise as a key pillar of their well-being, with noticeable improvements in patients’ day-to-day function, physical fitness, body composition, energy levels, emotional wellness, and overall physical activity levels.

*In the beginning ah I needed so much help […] I was unable to pull my own pants up. I was unable to cut my own toilet paper […] I’ve come a long way from that […] Now, I’m out walking on the trails and walking on the hills and making plans for the future, now that I know I will be able to.* 

(Patient no. 14, female, 68, glioblastoma).

Consistent with behaviour change research [34], those with more to gain from engaging in exercise (i.e., those who were engaging in less exercise at the outset of the study or higher burden of disease), tended to experience more profound benefits of exercise. For many, ACE-Neuro was a source of hope for wellness during a daunting time in their lives and gave them purpose to do something meaningful during the day. It also provided a sense of control in their cancer journey, control they felt they lost when they were first diagnosed with cancer. By building the habit of movement as part of ACE-Neuro, participants started to see themselves as exercisers (for the first time, or again) and felt that the program was not only a catalyst for exercise behaviour change, but other health habits such as nutrition and sleep. Participants also became more confident in their physical abilities and body image and became inspired to engage in activities beyond the formal exercise setting. With improved patient function and confidence, the burden of care experienced by caregivers was also lessened, leading to improved quality of life for both patients and caregivers.

*I’m starting to fit my original clothes […] If you look at this t-shirt… I deliberately wore it…I was wearing it on the first day I saw you [6 s pause]…look at it on me now. It was tight when I first started with you guys, and it’s not a small, you know? This is me under here, right?* 

(Patient no. 6, female, 42, glioblastoma).

The program was described as having an emphasis on community-building, a shared experience with others living with a similar diagnosis, positive reinforcement, and the “right” amount of encouragement to motivate exercise behaviour change. The people—other participants, the instructors, and volunteers—were what made ACE-Neuro feel like a community. Many participants also felt that the program offered a protected space to share thoughts and challenges and move in a way that felt welcome and supported. This culture of movement was especially valued by patients and caregivers as a way for reimagining what it means to be physically active whilst navigating the cancer continuum. Many participants were also surprised to discover deep emotional connections with each other, revealing the power of empathy and presence, despite the screen/online delivery.

*It might be just like the way that somebody, like looks back at the camera at you […] I could see [another participant] working so hard and [his caregiver] in the background, helping him so much. And then the one day that we talked about getting ready for good news and bad news, and he looked up at the camera, he looked at me and I thought, oh, [name of participant], we just got bad news. And I’m sorry. And it was really, that was really tough. And I just felt he looked right straight at me, like right into my heart […] I never expected that.* 

(Patient no. 14, female, 68, glioblastoma).

For the patients who only participated in one-on-one exercise (i.e., due to preference or need for more individualized support), they still felt connection to the larger neuro-oncology community, through the trained exercise specialists and knowledge that their peers were accessing and engaging with the same wellness resources.

*Having [this program] is a testament that everybody is working hard to improve, working hard to, uhm, build their strength but at the same time […] enjoying it as well and with the company of the support people and also your co-patients.* 

(Patient no. 4, female, 53, meningioma).

Ultimately, participants felt empowered in their health journeys, attributing the ACE-Neuro program as a meaningful part of their lives. The positive motivational climate instilled throughout delivery, from recruitment to study completion, optimized participant outcomes.

**Theme 4—The Resolution: Tailored Not Templated…The Ideal Program for Me** 

Each individual’s story of participating in ACE-Neuro reaches its conclusion with learning how the program was best tailored to their needs. Patients and caregivers described how the benefits of ACE-Neuro, from physical to psychosocial well-being, were supported by their own blueprint for exercising. A non-negotiable of this blueprint was providing participants with a choice for the format of delivery (i.e., online, in person, or hybrid and one-on-one or group sessions), number and intensity of sessions per week, type of exercise performed, and type of health coaching/wellness education integrated. Further, ensuring ACE-Neuro was available as a resource across all stages of the cancer continuum is important, allowing participants to choose when they want to enrol.

*Oh, it’s nice to have the option. Cause there were sometimes that… it would have been very difficult for me to attend on time those [in-person] classes. So, I was happy that it was available as ZOOM^TM^ because then it’s just a matter of setting up my space and moving from one room to the other.* 

(Patient no. 73, female, 63, oligodendroglioma).

Though some participants could only manage one session per week at points throughout the intervention (e.g., due to disease progression), they still wanted the option of two scheduled sessions, leaving room for other weekly commitments (e.g., appointments) whilst supporting accountability and building their exercise habit. Others would have liked to progress to three or even four times per week, especially since many were either retired or on short or long-term disability and were not working during the week. Participants all described how they thought that the intensity and length of sessions should vary based on the point they were in treatment, acute energy and fatigue levels, cancer-related symptoms, and goals, with exercise ranging from 15 to 90 min per session. Shorter duration sessions should be scheduled during low energy, high fatigue, or high symptom burden days. Longer duration sessions should be for the “better” days. Working towards 90-min sessions was important for those with fitness-related goals (e.g., wanting to increase maximal muscle strength). These differing preferences underscore the importance of acknowledging the variability of diagnoses, as well as participants’ stages of change in their journey to wellness and stage of cancer continuum—where those further into maintenance may seek more frequent and intense programming than those who are still seeking to find a feasible routine to adhere to [33].

*60 [minutes] was awesome. Unless I was having a bad day. I think there were a couple where we only did 30, because I just wasn’t feeling good in general like not exercise related, but like the day had been not a great day.* 

(Patient no. 65, female, 40, astrocytoma).

Many participants valued and felt they warranted one-on-one sessions, even when participating in group sessions. This feedback further supports the need for individualized interventions. While group settings provide social support and community building, the one-on-one setting was seen as critical for achieving personal goals, highlighting the value of tailored feedback and direct support.

*I love that, uhm, also there is a one-on-one because, uhm, when you have one-on-one, you know, at least they can monitor you more rather than having it in a class […] because we have different situations. But on one-on-one, it’s really specific based on your, uhm, case, let’s say your situation, which is really good.* 

(Patient no. 4, female, 53, meningioma).

Both formats were also seen as valuable for fostering self-efficacy, a key construct across all theories of exercise behaviour change. To serve participants’ varied and dynamic needs, a good quality program must offer a menu of choices and leverage participant autonomy and self-identified priorities. The tailoring of delivery should be based on the timing of treatment (i.e., on or off active treatment), acute energy and fatigue, cancer-related side effects and deficits, and movement history, confidence, abilities, and preferences. From a behaviour change lens, recognizing that adhering to and maintaining exercise behaviour is not a linear and “one size fits all” approach is important—it is a dynamic and individualized process that requires ongoing adaptation and tailored support instead.

**Theme 5—The Epilogue: Key Factors for Sustained Delivery** 

Looking ahead, many participants intend to continue exercising and building their wellness routine. Many also shared their perspectives of an ACE-Neuro “wish list” for future success in the scalability and implementation of programming. First, patients and caregivers spoke to their hope that additional funding can be secured to continue the delivery of initial programming at zero cost to patients, ultimately supporting their motivation/”buy-in” to access exercise resources. Participants also wanted to see continued/maintenance programming offered at a minimal cost to support sustained exercise engagement and ongoing health benefits after the initial/baseline offering.

*I hope that you can manage getting the funding for it, because it would be a shame for people to not enter because it wasn’t free, and therefore they never got started. A lot of people start, they do the free part, and then say, ‘Yeah, I like that… let’s continue.’ […] If you have to pay at the beginning, people aren’t gonna join. And you’re not gonna get it continuing. So, it’s very helpful to have it free and open to those at the beginning.* 

(Patient no. 48, female, 63, glioblastoma).

Second, patients and caregivers described the need for implementing multiple location options for in-person exercise, accessible community gyms, continuing to train exercise professionals to work with the cancer population, and including additional equipment and education topics.

*If money wasn’t an issue, I think it would be brilliant to be able to have multiple locations in the city where cancer patients can access gyms that are accessible and are able to be used by people who are high risk.* 

(Caregiver no. 59, female, spouse).

To that end, the need for ACE-Neuro to be integrated as a clinical program and prioritized by the cancer care system was expressed as a major gap in current care. Participants described ACE-Neuro as being disjointed from the rest of cancer care, though it is seen as an equally important component of supportive cancer care. To champion this integration and increased capacity for programming, participants spoke to the level of support needed. Beyond the individual, concerted efforts are needed from caregivers, exercise specialists, healthcare providers, and the larger cancer care system to support access to wellness resources. The participants who were several years post-diagnosis wished ACE-Neuro had been available earlier and are thus hopeful that programming will continue to be accessible to prevent delayed exercise support.

*I feel like this this should be part of all the programs at [the cancer centre]. There’s psychosocial, there’s dietitians, and earlier on in my cancer journey I was sent to either of those […] I’ve also been sent to physio at one point by my family doctor, so I know, like, you know, I have experienced that. So, I really think the exercise part, if it could be incorporated, especially if they cancer center there, should I think it should be part of the package.* 

(Patient no. 65, female, 40, astrocytoma).

Participants felt inspired to advocate for other individuals living with and beyond brain cancer to participate in neuro-oncology exercise programming. Their own experiences with ACE-Neuro became a source for inspiring others. To continue advancing the field of exercise and neuro-oncology, a multi-level approach is needed to achieve the vision of an improved and successfully sustained ACE-Neuro program.

## 4. Discussion

### 4.1. Overall Summary

The purpose of this study was to explore neuro-oncology patient and caregiver experiences participating in the Alberta Cancer Exercise-Neuro-Oncology (i.e., ACE-Neuro) program. Our work depicts the story of patients and caregivers in their journey finding ACE-Neuro, navigating obstacles and facilitators to participation, experiencing the benefits of movement in their wellness, needing a tailored blueprint for participation, and key factors for sustained successful delivery. A novel photo elicitation approach coupled with semi-structured interviews, along with engaging a patient partner throughout analysis, was used to better understand these experiences.

Exploring the role of exercise in the brain tumour population is still vastly understudied. Though qualitative approaches have been widely used in exercise oncology, the literature is limited for examining the experience of exercise among individuals living with and beyond brain cancer. Where is the focus on exercise as a supportive neuro-oncology care resource? Our work thus begins to address this considerable research gap by building upon the qualitative evidence base in exercise and neuro-oncology. It is clear from our findings that patients and caregivers value the opportunity to exercise, experience direct benefits of exercise on outcomes, and see a future of exercise integrated within neuro-oncology care. These results are consistent with other qualitative work conducted with high-grade-glioma patients and their caregivers participating in exercise during chemoradiotherapy [18] and across other underserved populations [35,36,37]. Our findings have enriched this limited knowledge base for understanding patient and caregiver needs for implementing exercise interventions that are tailored to individuals’ needs. Further, the needs expressed by patients and caregivers for additional systemic and policy-level support within our work are consistent with the social ecological model of change, which details the multiple levels of support needed to support exercise behaviour change [38]. The development of tailored and patient-centred neuro-oncology exercise programming plus system-level support (e.g., for referral) will ensure that the benefits of exercise are available to all patients and caregivers. To support program implementation, considering behaviour change theories, models, and frameworks is valuable.

### 4.2. Study Strengths

A notable strength of this work was employing a family-centred approach to interviews, wherein spouses, children, and parents (i.e., anyone who identified as a caregiver) were welcome to participate. Including caregivers was helpful for capturing the breadth of experiences as well as supporting patients who had neuro-oncology-specific challenges to participating in interviews (e.g., memory, cognitive, communication barriers). The analysis day itself was instrumental for developing themes in a unique format that told a story of patient experiences. Key factors that facilitated engaging the patient as a partner in research included (1) building meaningful relationships with the patient partner during the study period/intervention delivery, (2) treating the patient partner as an equal research team member and giving them the same opportunities to understand and refine the research question, review the data, and have an equal role in analysis, and (3) hosting the analysis day in-person with ample space and time for discussion (Supplementary File S3). Finally, the use of multiple methods for understanding the patient and caregiver experience (e.g., interviews augmented with photo elicitation and patient partner engagement) allowed for a profound investigation as well as created an inclusive space for patients and caregivers to share their voices.

### 4.3. Study Limitations and Opportunities for Future Qualitative Work

There was no patient engagement during the development of the interview guide. Future work would benefit from a patient partner role when identifying what questions to ask during interviews. While this study addressed an underserved patient group, the sample were predominantly white and of high socioeconomic status. Further, only English-speaking patients were included as the research team was only able to conduct interviews in English and program delivery did not have translation services. There thus remains inequity in access within this underserved neuro-oncology population, and future work must address diversity in participants. Research is also needed to interview patients who chose (1) not to enrol into programming and (2) to withdraw from programming. Finally, Table 3 presents a summary of implementation needs for future work in neuro-oncology exercise research.

## 5. Conclusions

The findings from our qualitative photo elicitation study clearly highlight the need for the systematic inclusion of exercise across the neuro-oncology care continuum. Continuing to work with patient partners at multiple levels of the research process will facilitate understanding of broader implementation needs to ensure the sustainable delivery of accessible, tailored, and patient-centred neuro-oncology programming.

## Figures and Tables

**Table 1:** Patient clinical characteristics and demographics (*n* = 28).

Clinical Characteristics and Demographics	Number of Participants (%)
Self-Identified Sex	
Male	12 (42.9%)
Female	16 (57.1%)
Age: Mean ± SD, years	51.4 ± 11.7 (range: 32–81)
Time Since Diagnosis: Mean ± SD, months	58.5 ± 84.1 (range: 1–290)
Type of Primary Brain Tumour	
Glioblastoma	9 (32.1%)
Oligodendroglioma	3 (10.7%)
Astrocytoma	5 (17.9%)
Meningioma	4 (14.3%)
Germinoma	1 (3.6%)
Medulloblastoma	1 (3.6%)
Presumed Glioma	1 (3.6%)
Malignant Glioma Not Otherwise Specified	1 (3.6%)
Craniopharyngioma	2 (7.1%)
Pineocytoma	1 (3.6%)
Histologic Grade	
1	2 (7.1%)
2	4 (14.3%)
3	7 (25%)
4	10 (35.7%)
Unknown	5 (17.9%)
Treatment Status	
Pre-Treatment *	1 (3.6%)
On Treatment	11 (39.3%)
Off Treatment	16 (57.1%)
Treatment Type	
Surgery Alone	1 (3.6%)
Surgery + Radiation	5 (17.9%)
Surgery + Chemoradiation	2 (7.1%)
Surgery + Radiation + Adjuvant Chemotherapy	6 (21.4%)
Surgery + Chemoradiation + Adjuvant Chemotherapy	13 (46.4%)
Chemoradiation + Adjuvant Chemotherapy	1 (3.6%)
Smoking Status	
Never Smoked	15 (53.6%)
Ex-Smoker	11(39.3%)
Occasional Smoker	1 (3.6%)
Regular Smoker	1 (3.6%)
Alcohol Drinking Status	
Never Drinker	6 (21.4%)
Ex-Drinker	5 (17.9%)
Occasional Drinker	12 (42.9%)
Social Drinker	4 (14.3%)
Regular Drinker	1 (3.6%)
Marital Status	
Never Married	4 (14.3%)
Married	22 (78.6%)
Separated	1 (3.6%)
Divorced	1 (3.6%)
Education	
Some High School	2 (7.1%)
Completed High School	2 (7.1%)
Some University/College	3 (10.7%)
Completed University/College	13 (46.4%)
Some Graduate School	1 (3.6%)
Completed Graduate School	7 (25%)
Annual Family Income, CAD	
<CAD 20,000	3 (10.7%)
CAD 20,000–CAD 39,999	3 (10.7%)
CAD 40,000–CAD 59,999	2 (7.1%)
CAD 60,000–CAD 79,999	1 (3.6%)
CAD 80,000–CAD 99,999	1 (3.6%)
>CAD 100,000	9 (32.1%)
Prefer not to answer	9 (32.1%)
Employment Status	
Short-Term Disability	2 (7.1%)
Long-Term Disability	12 (42.9%)
Retired	6 (21.4%)
Part-Time	1 (3.6%)
Homemaker	2 (7.1%)
Full-Time	3 (10.7%)
Unemployed	2 (7.1%)
Self-Identified Racial Background **	
East Asian	1 (3.6%)
Indigenous	2 (7.1%)
South Asian	2 (7.1%)
Southeast Asian	1 (3.6%)
White	22 (78.6%)

* Pre-treatment is before surgery, chemotherapy, and/or radiation. ** Reported based on the Canadian Institute for Health Information’s Guidance on the Use of Standards for Race-Based and Indigenous Identity Data Collection and Health Reporting in Canada [32].

**Table 2:** Caregiver characteristics (*n* = 9).

Caregiver ID	Sex	Relationship to Patient	Interview Mode
8	Female	Spouse	Individual (without patient)
17	Male	Spouse	Dyadic (with patient)
36	Female	Spouse	Dyadic (with patient)
37	Female	Child	Dyadic (with patient)
40	Female	Spouse	Dyadic (with patient)
43	Male	Spouse	Dyadic (with patient)
56	Male	Spouse	Dyadic (with patient)
59	Female	Spouse	Dyadic (with patient)
61	Female	Parent	Dyadic (with patient)

**Table 3:** Summary of findings: key insights and research needs for implementing neuro-oncology exercise programming.

Theme	Key Insights	Exemplary Quote	Research Needs	Markers of Success
1. The Exposition: I Have Cancer…Now What?	Living with brain cancer is challenging—what can be done to support patient wellness?	“*After the tumour, I haven’t relearned how to do everything again because it’s like being born.*” (Patient no. 17, female, 47, medulloblastoma).	Investigate the relationship between the physical and psychosocial experience with brain cancer and seeking wellness resources. Explore effective communication strategies for providing information on resources to newly diagnosed patients.	Centralized and patient-friendly information platform for exercise oncology/wellness resources.
2. The Rising Action: Trials and Triumphs of Participation.	What hinders and supports participation in neuro-oncology exercise programming?	“*I would have something to look forward to each week, two days a week. It was really cool because it gave me that incentive to keep working out.*” (Patient no. 59, male, 47, germinoma).	Develop enhanced referral pathways to exercise programming. Develop strategies to address barriers and facilitators to participation in programming (e.g., strategies to facilitate completion of measures (e.g., patient-reported outcomes) that are inclusive of disease-specific limitations).	Increased participation/enrollment of neuro-oncology patients in programming. Referral pathways integrated into electronic medical records. Increased completion rates of measures.
3. The Pivotal Moment: It’s More Than Exercise.	What does participating in neuro-oncology exercise programming mean to the patient and caregiver?	“*I got my trophy.* (Patient no. 56, female, 39, astrocytoma). *The dumbbell is your trophy?* (JTD, first author). *Yeah.*” (Patient no. 56, female, 39, astrocytoma).	Investigate the relationship between exercise and treatment efficacy and treatment-related side-effects (e.g., thromboembolic complications). Investigate constructs of behaviour change (e.g., self-efficacy) in relation to exercise adherence and maintenance.	Increased number of clinical trials and effectiveness-implementation trials in exercise and neuro-oncology. Improved patient outcomes. Improved understanding of behaviour change in neuro-oncology.
4. The Resolution: Tailored Not Templated…The Ideal Program for Me.	One size does not fit all—how do we tailor programming to meet the needs of an individual?	“*The coaches identify with each person as to what they need because everybody is going to be different based on where they’re at from their recovery.*” (Patient no. 1, female, 58, meningioma).	*Compare* feasibility and effectiveness of tailored exercise prescription components (frequency, intensity, time, type, format of delivery) across cancer continuum (i.e., pre-, during, and post-treatment).	Development of evidence-based neuro-oncology-specific exercise guidelines.
5. The Epilogue: Key Factors for Sustained Delivery.	How do we maintain successful implementation and increase capacity for programming?	“*I just I hope that there is continued funding so that other people can have the same access.*” (Patient no. 65, female, 40, astrocytoma).	Integrate exercise programming into standard neuro-oncology care via a transdisciplinary and multi-level approach.	System-level support and funding instilled for exercise-oncology programming. Continued opportunity for programming (currently there is no funding to sustain delivery). Additional venues/locations for program delivery.

## Data Availability

Data will be made available upon request.

## Supplementary Materials

The following supporting information can be downloaded at: https://www.mdpi.com/article/10.3390/curroncol32020111/s1, File S1: Photo Elicitation Protocol; File S2: Study Interview Guide; File S3: Overview of ACE-Neuro Qualitative Analysis Day; File S4: Additional Exemplary Quotes.
