# Supplementary material for: “I Got My Trophy”: The Story of Implementing a Neuro-Oncology Exercise Program from the Patient and Caregiver Lens—A Qualitative Study
Source: Curr Oncol. 2025 Feb 16;32(2):111. doi: 10.3390/curroncol32020111 (PMC11853919; doi:10.3390/curroncol32020111)
Supplement: Supplementary file 1 [file curroncol-32-00111-s001.zip › File S1. Photo Elicitation Protocol.pdf]

The ACE-Neuro Experience – Through Your Eyes

Tell Us About Your ACE-Neuro Experience

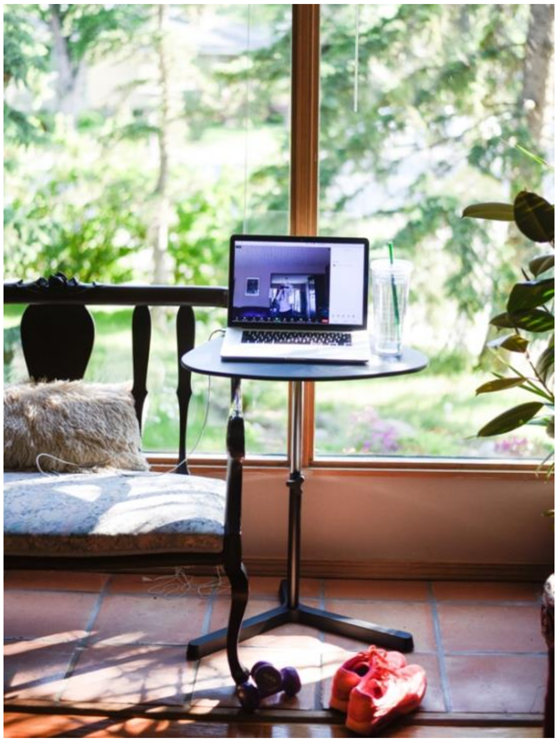

As part of ACE-Neuro, we would like to capture your experience with the program, through your photos and your voice. The process is simple – capture images of your ACE-Neuro! These can be of yourself exercising, of your exercise setting, your equipment, with your instructor, with your family…whatever ACE-Neuro represents for YOU!

The study coordinator may also visit your virtual program (individual or class) to take photos (only for those that provide consent). These photos will be used in our ACE-Neuro post-program interviews to help learn more about your experience

Photos can be taken by your camera or cellphone. If you do not have either, we will provide a disposable camera for your use over the next few weeks. Please let us know if you will need a disposable camera.

How to Send Us Your Photos

You can send your photos to the research team in one of two ways:

<u>Option #1</u>: e-mail your photos to the study coordinator at: [redacted]

<u>Option #2</u>: send your photos via the end-to-end encrypted app, **Signal**. This ensures that the photos are sent securely, and this has been approved in our ethics (ID: *HREBA.CC-20-0322*).

The study team will store the photos only on a secure file in our lab, and the photos will only be used within our subsequent interviews, and will only be shared with your permission.

To use the Signal App, you may download it to either your phone or desktop, by following the instructions here: https://signal.org/en/download/

Once downloaded, please follow the instructions presented on the next page.

1

Once you have downloaded the app, go to your home page and create a new message/chat by clicking the pencil icon at the top right corner of your screen

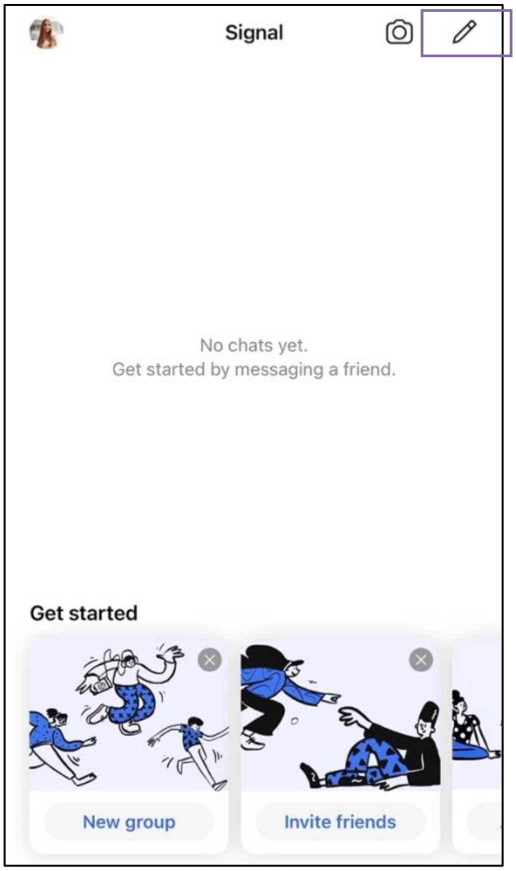

2

Select “Find by Phone Number”

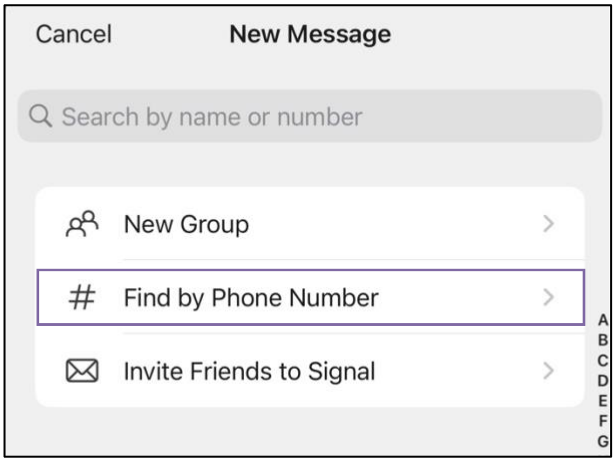

3

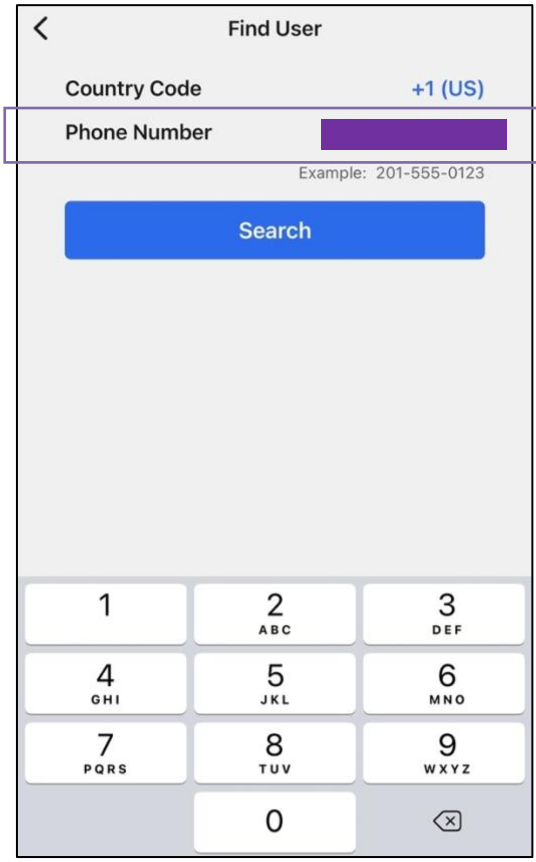

Type the research team’s phone number [redacted] and press “search.” You will find our profile and will be ready to send her your photos!

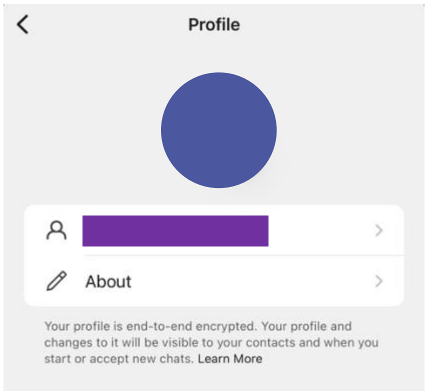

Photo-Taking Tips

Please see below for a few helpful tips for capturing your photos:

- **Be creative and have FUN!** Some photo type ideas include:
  - **Screenshot** – take a screenshot of your ZOOM™ exercise session.
  - **Selfie** – take a photo of yourself before, during, or after your session.
  - **Have someone take a photo of you** – perhaps in front of your exercise set-up, or of you doing your favourite exercise.
  - **Self-timer** – many phones have this feature. You could setup your camera/phone on a high surface and snap some creative photos.

- **Ensure your camera lens is clean** – this is for both your camera and phone. Often, our phone camera lenses get dirty from everyday use – give your phone lens a quick wipe with glass cleaner, a microfiber cloth, or soft cotton material (like your t-shirt).

- **Ensure your space is well-lit**. If your exercise space is in a low-light area, don't stress! Try using different lights to brighten your space. If your photo turns out dark, don't stress! We (or you!) can quickly edit it to bring out the shadows and brightness! Or even leave it dark for a "moodier" snapshot!

- **Try playing around with different angles**! Try taking photos from up high, down low, or somewhere in between!

- Send your images in **JPEG format** if possible.

Remember, there is no "right" or "wrong!" Every photo is unique and beautiful in its own way!!
We are excited to see what you come up with!
