# Supplementary material for: “I Got My Trophy”: The Story of Implementing a Neuro-Oncology Exercise Program from the Patient and Caregiver Lens—A Qualitative Study
Source: Curr Oncol. 2025 Feb 16;32(2):111. doi: 10.3390/curroncol32020111 (PMC11853919; doi:10.3390/curroncol32020111)
Supplement: Supplementary file 1 [file curroncol-32-00111-s001.zip › File S2. Study Interview Guide.pdf]

**File S2 –** Study Interview Guide – Daun et al.

Patient Interview Guide

- Interview #1 – Baseline Assessment……………………………………………………………………2
- Interview #2 – Post-Program Completion…………………………………………………………..3

Caregiver Interview Guide……………………………………………………………………………………….5

## INTERVIEW #1 – DURING BASELINE ASSESSMENT OR SHORTLY AFTER (15 MINS)

## INTERVIEW QUESTIONS

1. I'd like to begin today's interview by talking about the recruitment for this study. If you remember, you were introduced to this study by your healthcare provider. Next, I phoned you to further explain what the study would entail. Could you please tell me about your experience with this recruitment process?
   Probes:
   - How was the timing with respect to your diagnosis and treatment?

2. If you remember, you came to see us for an in-person appointment at the Holy Cross Hospital before starting the exercise program. Could you please tell me about your experience with this appointment?
   Probes:
   - How did you find the duration of the appointment?
   - How did you find the assessments performed during the appointment?

*IF PARTICIPATING IN PHOTO ELICITATION*

- I'm going to show you a photo of the appointment room and set-up. When you look at this photo, how do you feel? What comes to mind?
- How did you feel before, during, and after the appointment?

3. Is there anything else you'd like to share about your experience with the recruitment process for this study?
   Probes:
   - Ease/difficulties?

## INTERVIEW # 2 – AT 12 WEEKS/POST PROGRAM (30-45 MINS)

### INTERVIEW QUESTIONS

**1.** I'd like to begin our interview by talking about physical activity. Would you please tell me about your physical activities before and after your cancer diagnosis?
Probes:
- How has your treatment affected your activity level?
- What role does physical activity play in your recovery?

**2.** Now I'd like to ask you some questions about your experience with the exercise program. Would you please tell me about your experience in the 12-week exercise program?
Probes:
- How did you find the sessions?
  - How did you find the frequency of sessions?
  - How did you find the intensity sessions?
  - How did you find the time of each session?
  - How did you find the type of exercise included in sessions?
  - How was the timing w/ respect to cancer journey?
- How did you find the safety of the program?
- How did you find the role of the exercise specialist?
- How did you find the role of the moderator?
- How did you find the format of 1 on 1 and group classes?
- How did you find the format of online sessions?
- How was your experience with the tailored programming?
- How did you find the education received as part of the program?
- What was your experience with the health coaching?
  - How did your health coach support your wellness?
  - How did you find 1 on 1 versus group-based coaching?

**3.** We had a number of assessments as part of the ACE-Neuro study. I am going to ask you about each one:

a) Would you please tell me how you found the online questionnaires, completed via REDCap?
Probes:
- What was your experience with the duration to complete the questionnaires?
- How did you find the questions we asked?

a) Would you please tell me how you found the fitness assessments, completed via Zoom?
Probes:
- What was your experience with the duration to complete the assessment?
- How did you find the tests included in the assessment?

b) Would you please tell me how you found the usage of the Garmin Activity Tracker throughout the exercise program?
Probes:
- What was your experience wearing it across the 12 weeks?

- What was your experience tracking your activity and steps?
- What was your experience setting up the tracker?

c) To date, we completed these assessments at two timepoints: before and after the exercise program. Would you please tell me how you felt about the timing of these?
Probes:
- Do you have any examples of what you've liked or found difficult with these timepoints?

d) Would please tell me if there is anything about the assessments that you'd like to see changed?

**4.** *IF PARTICIPANT CONSENTED TO PHOTO ELICITATION*

If you remember, part of this study included taking photos, and having photos taken of your experience in ACE-Neuro. I'm going to show you a few photos now.

Would you please describe what is happening in the photo(s)?
Would you please tell me what comes to mind when I show you these photos?
Probes:
- How does this photo make you feel?
- Why did you choose to take this photo?
- What do you think about this photo?
- What does this photo represent to you?
- How does this photo relate to your diagnosis, treatment, and/or recovery?
- Is there a particular thought/memory that comes to mind when looking at this photo?
- What would you tell a newly-diagnosed patient about this program when looking at this photo?
- Looking at this photo, and thinking of your time in the ACE-Neuro program, how would you describe your experience in three words?
- How does this photo portray/represent your experience in the ACE-Neuro program?

**5.** How would you describe the role of your healthcare team in ACE-Neuro?
Probes:
- How did your healthcare team support your ACE-Neuro involvement?

**6.** How would you describe the role of your caregiver in ACE-Neuro?
Probes:
- How did your caregiver support your ACE-Neuro involvement?

**7.** Is there anything you would like to see done differently in this study?
**8.** Is there anything else you would like to tell me about your experience in this study?

## CAREGIVER INTERVIEW GUIDE

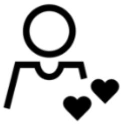

### INTERVIEW QUESTIONS

**1.** I'd like to begin by talking about exercise. From your perspective, what is the role of exercise for individuals affected by cancer?

Probes:
-What is the impact of exercise on well-being?
-What is the role of exercise before, during, or after treatment?

**2.** Would you please tell me about how you think the ACE-Neuro study impacted (INSERT NAME/RELATIONSHIP TO PATIENT)?

Probes:
-What benefits/challenges did you notice?
-What was it like seeing (PT NAME) participating in ACE-Neuro?
-What is your perspective on the safety of the program?

**3.** Would you please tell me about what your role looked like supporting (INSERT NAME/RELATIONSHIP TO PATIENT) during their participation in ACE-Neuro?

Probes:
-How did (PT NAME) participation in ACE-Neuro affect you in your caregiver duties/role?

**4.** What has it been like trying to take care of yourself (i.e., pay attention to your own health) while you have been a caregiver to your family member with cancer?

Probes:
-What did you have to adapt/change in your personal life while caring for (PT NAME)
-How did (PT NAME) participation in ACE-Neuro affect your well-being?

**5.** Based on your caregiver role, how do you think we could improve ACE-Neuro?

Probes:
-What are some things we could do differently/better?

**6.** What role do you feel the exercise professional plays in the care of (PT NAME)?

Probes:
-How did the exercise professional support (PT NAME) during their cancer journey?

**7.** What role do you feel the healthcare provider plays in supporting (PT NAME) to exercise?

Probes:
-How did (PT NAME) healthcare team support their participation in ACE-Neuro?

**8.** Is there anything else you would like to share about your and (PT NAME) or your experience in ACE-Neuro?
