# Supplementary material for: “I Got My Trophy”: The Story of Implementing a Neuro-Oncology Exercise Program from the Patient and Caregiver Lens—A Qualitative Study
Source: Curr Oncol. 2025 Feb 16;32(2):111. doi: 10.3390/curroncol32020111 (PMC11853919; doi:10.3390/curroncol32020111)
Supplement: Supplementary file 1 [file curroncol-32-00111-s001.zip › File S3. Overview of ACE-Neuro Qualitative Analysis Day.pdf]

ACE-Neuro Qualitative Analysis Day

STEP 1

REVIEW RESEARCH QUESTION: 30 MIN

- Review purpose of the study.
- Ensure that the patient partner and analysis team are clear on the research process.

STEP 2

ORGANIZE AND VISUALIZE CODES: 60 MIN

- Coding is completed prior to the analysis day.
- Write out all finalized codes on large sticky notes.

STEP 3

GROUP CODES: 120 MIN

- Map codes together based on similar ideas.
- Physically move sticky notes into groups.
- Deep reflection, interpretation, and discussion of code groupings.

Breaks included throughout the day

STEP 4

CREATE INITIAL THEMES: 90 MIN

- The story of ACE-Neuro is created.
- Analogy of a story in five acts is conceptualized by the patient partner to articulate the results.
- A story plot diagram is drawn and detailed with five themes.
- Critical review of themes.

STEP 5

REVIEW AND FINALIZE THEMES: 60 MIN

- Refine each theme.
- Detail a brief description for each theme.
- Share results with co-authors/collaborators.

UNIVERSITY OF CALGARY
FACULTY OF KINESIOLOGY
Health and Wellness Lab

ACE-Neuro Qualitative Analysis Day

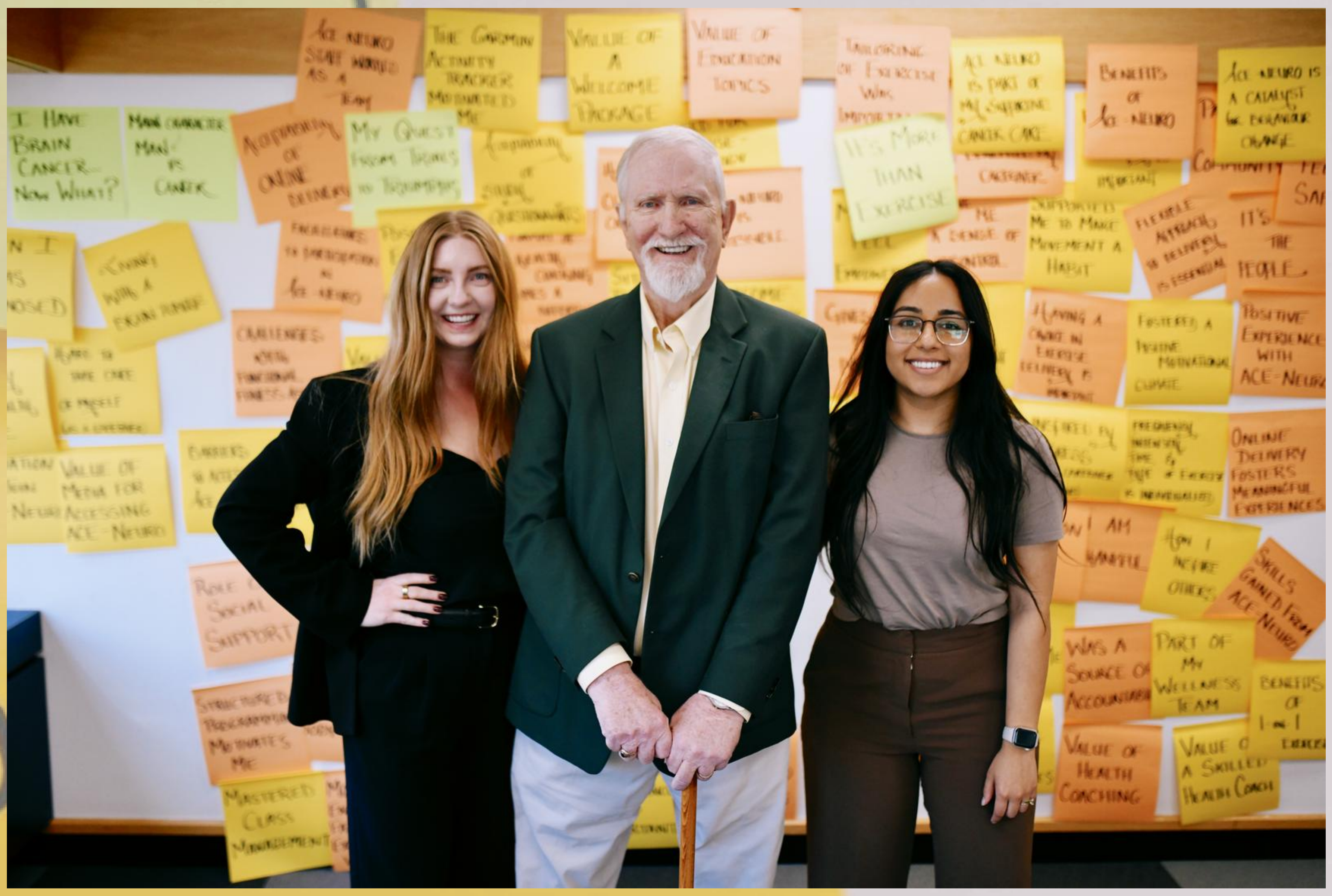

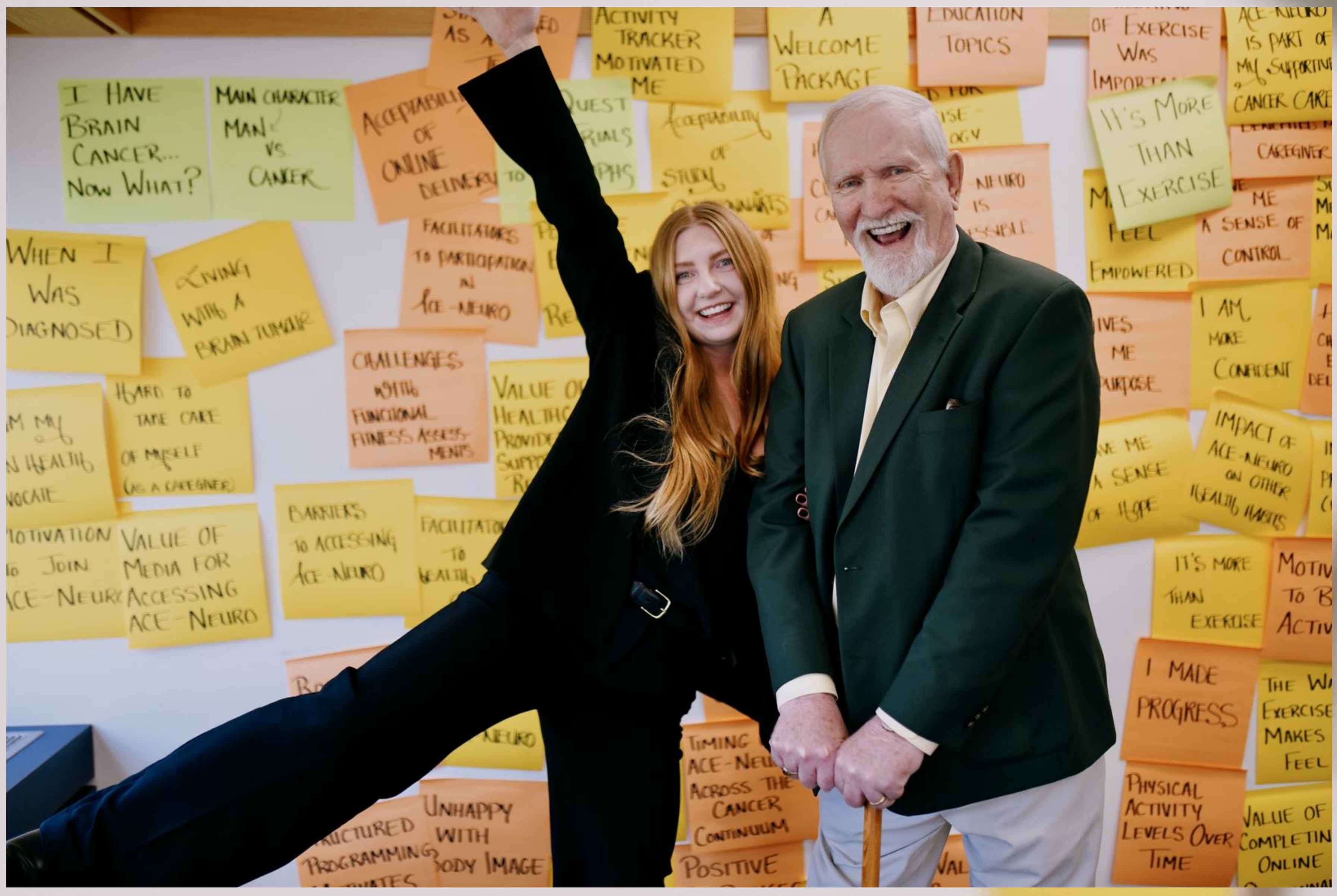

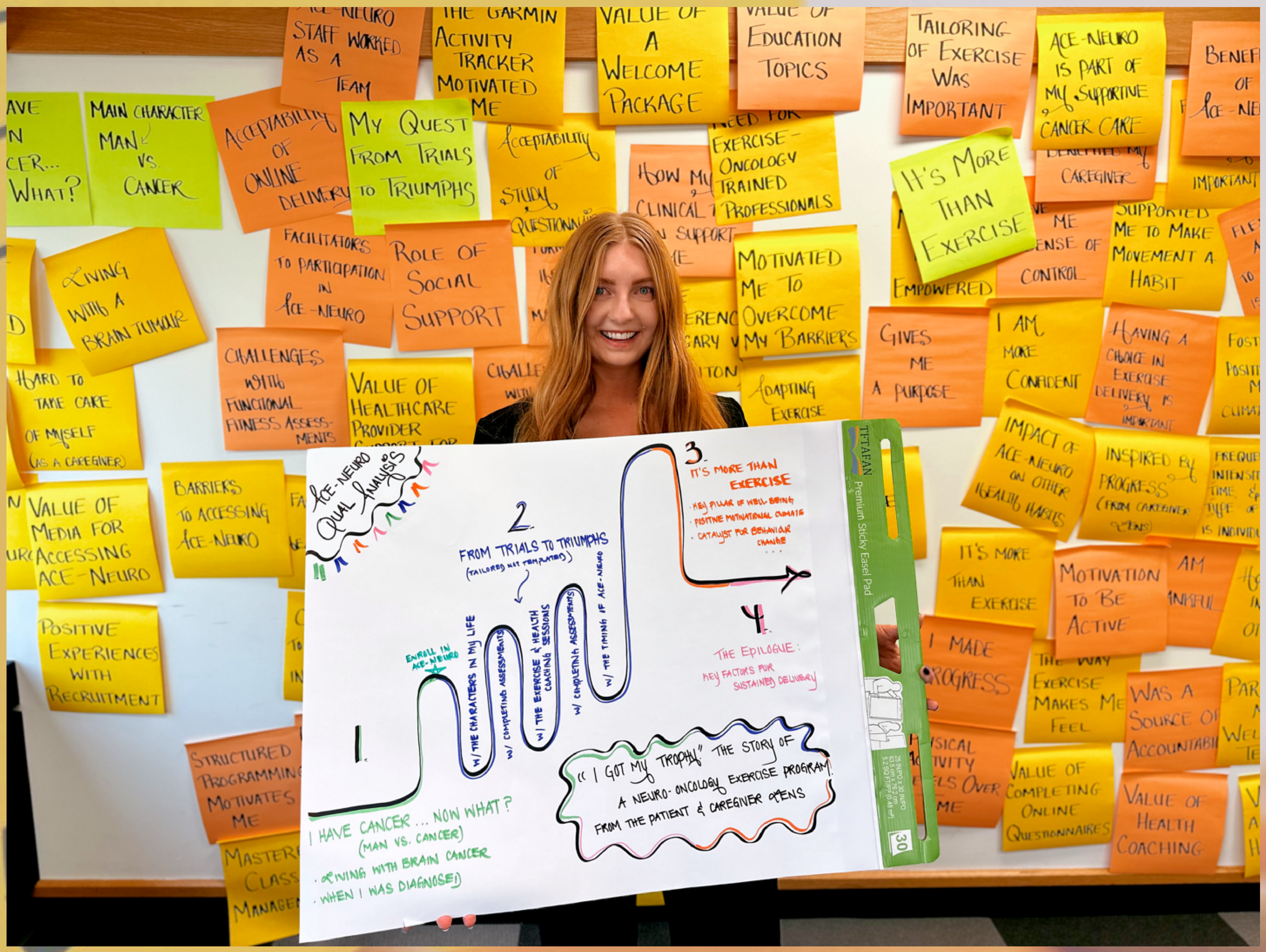

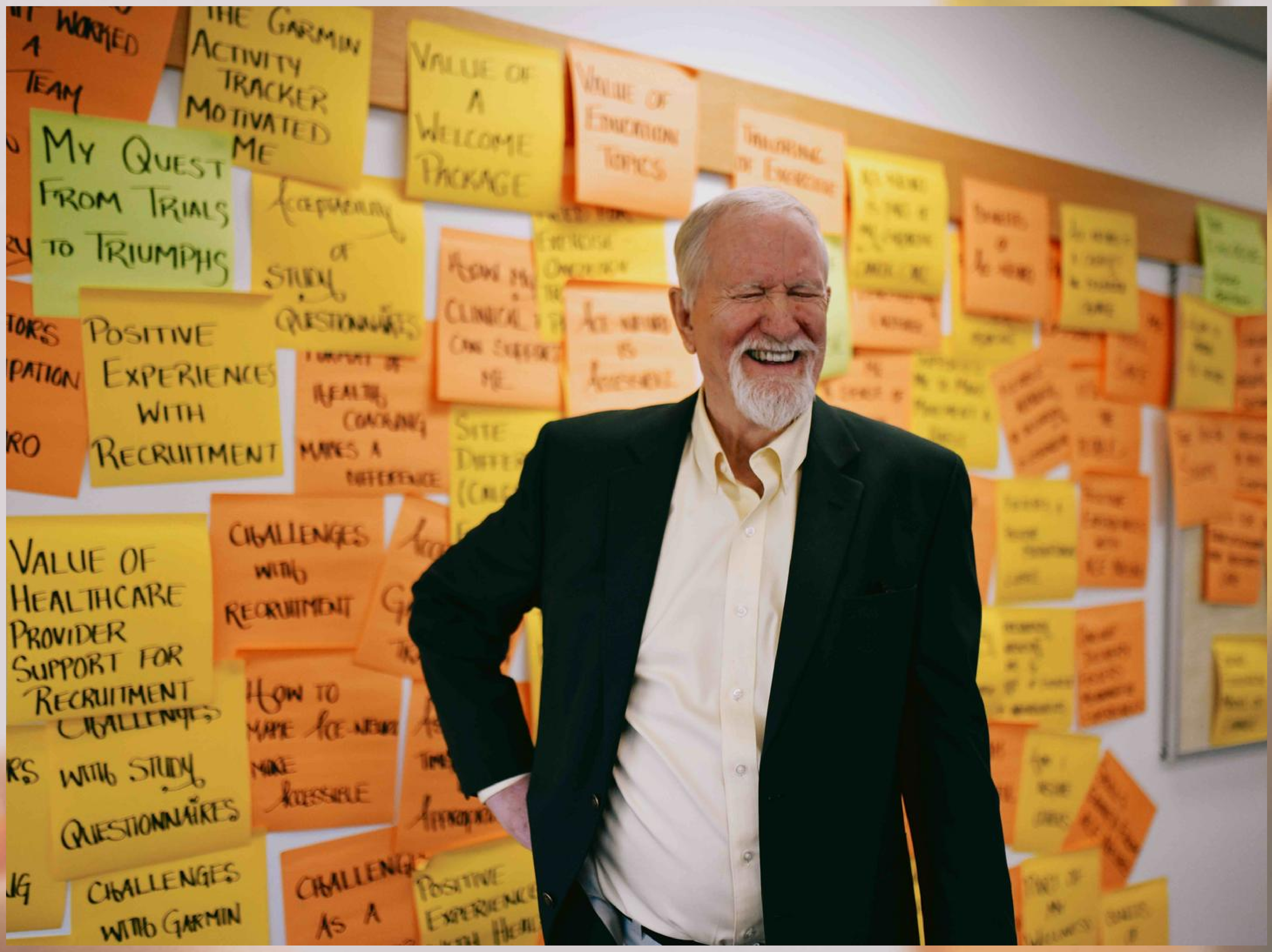

Photographs: Emma McLaughlin and Julia Daun

Watch the Analysis Day Here:

UNIVERSITY OF CALGARY
FACULTY OF KINESIOLOGY
Health and Wellness Lab
