# Supplementary material for: “I Got My Trophy”: The Story of Implementing a Neuro-Oncology Exercise Program from the Patient and Caregiver Lens—A Qualitative Study
Source: Curr Oncol. 2025 Feb 16;32(2):111. doi: 10.3390/curroncol32020111 (PMC11853919; doi:10.3390/curroncol32020111)
Supplement: Supplementary file 1 [file curroncol-32-00111-s001.zip › File S4. Additional Exemplary Quotes.pdf]

File S4 – Additional Exemplary Quotes – Daun et al.

**1. The Exposition**: I Have Cancer…Now What?	**2. The Rising Action**: Trials and Triumphs of Participation	**3. The Pivotal Moment**: It's More Than Exercise	**4. The Resolution**: Tailored Not Templated…The Ideal Program for Me	**5. The Epilogue**: Key Factors for Sustained Delivery
Imagine, that's an accomplishment. Just getting down on one knee and getting up again. If somebody told me that just a few years ago, I would never believe that. […] You know, I in fact, all together, I find it hard to believe the situation I'm in. My brain knows what I want to do […] if I know what I want to do, how come the brain doesn't, you know? Patient no. 36, male, 83, meningioma).<br><br>*After the tumour, I haven't relearned how to do everything again because it's like being born.* (Patient no. 17, female, 47, medulloblastoma).<br><br>*If there'd been any other way [to be recruited], I probably wouldn't bother […] Part of why I joined was because the next time I had my [neuro-oncology] appointment, I could actually say I was willing to join ACE […] I've been going to the [cancer centre] now for 11 years so I know uhm the doctors and the nurse practitioners very well so you know, I guess in the back of my mind…we kind of want to make them happy.* Patient no. 63, female, glioblastoma).	*I was incredible when I get what into the room and you guys took the assessment on me and then you gave me my equipment and everything and I got home and got it all set up and everything I felt… 'oh, I'm in a group now, I'm in a club, I'm going to be supported, I'm going to go move ahead with this rehab, and I'm going to, you know… start working on my goals and start checking things off.' So, yeah it just felt terrific.* (Patient no. 14, female, 68, glioblastoma).<br><br>*I looked forward to it every week. Like we would sit down and figure out when the workout would be… like what days, what times. And then I would have something to look forward to each week, two days a week. It was really cool because it gave me that incentive to keep working out.* (Patient no. 59, male, 47, germinoma).<br><br>*I found [the questionnaires] a bit long, and also the choices were uhm a lot of the choices were never, once a week, once a month, and something else. I don't think those are the right choices because it's not never or once a week. It's never or sometimes.* (Patient no. 48, female, 63, glioblastoma).	*By looking at this photo […] like, look at her face… she's looking happy […] if you didn't tell me that this is her left hand, I would never believe it. These photos mean a lot to me because like, I never expected that she will raise her left hand with a dumbbell like this […] that's a really good achievement […] this this will keep her motivated, that 'yes I can do it'. It's something that's truly… this is amazing…it's unbelievable.* (Caregiver no. 56, male, spouse).<br><br>*I got my trophy.* (Patient no. 56, female, 39, astrocytoma).<br>*The dumbbell is your trophy?* (JTD, first author).<br>*Yeah.* (Patient no. 56, female, 39, astrocytoma).<br><br>*Now that I've participated in the exercise program, I learned how to ride my bike again. So, I am riding my bike. Just walking a bit but that's my knee, that's not me. I'm golfing more. I'm going to go swimming now. I'm just moving forward, yes.* (Patient no. 1, female, 58, meningioma).<br><br>*To get out and be more active. Stop the sugar intake. Drink a lot more water. Not a little more, a lot more water […] I only sleep good after my work outs. Uhm, but it's made me think more of, hey I shouldn't drink 3 iced caps a day, you know and 1 liter of Pepsi. Especially if we wanna do the exercise, lose the weight that you want, so you can do the other stuff.* (Patient no. 58, male, 32, astrocytoma).	*[The instructor was] very good at knowing what would work and what wouldn't, and if something just didn't work […] you had like a plethora of different things that we could try […] with cancer, that made it very difficult to uhm, read what your body was gonna be able to do versus what was available to you. But you guys made it very easy to have a lot of availability, no matter what your body was open for.* (Patient no. 52, female, 33, astrocytoma).<br><br>*I couldn't do anything after chemo. I literally thought I was going to die from chemo. There was 1 – 2 mornings I woke up and I was like, this is it. I was going to die today. I honestly thought that. Chemo was horrible. Radiation wasn't bad… I probably could've worked out. I think I did work out during radiation. (Patient no. 59, male, 47, germinoma).*<br><br>*I did Tuesdays and Thursdays. And then, I think Thursdays turned into group […] I could have done another day…I maybe would have wanted to see a third day […] maybe halfway, at 6-weeks. (Patient no. 1, female, 58, meningioma).*	*I can't think of anything that wouldn't be such a huge departure. I mean, I know it's a study. Sometimes I come to the conclusion like, I wish they had a little bit more stuff available for dietary uhm information like contacting dietitians like I went through and have one now, who helped me. But I was just wondering if there was some way that we could do it better.* (Patient no. 69, male, 56, pharyngeal cranial cyst).<br><br>*I just I hope that there is continued funding so that other people can have the same access.* (Patient no. 65, female, 40, astrocytoma).<br><br>*The hospitals […] do a good job of leveling you off and preventing you from crashing. But then you're leveled off and you need something to get you back up to where you're living, and that's where courses like or programs like this enter into it and sometimes, you know, it'd be nice if the hospitals could push you a little bit on the way out.* (Patient no. 40, male, 66, meningioma).
